# Actin polymerization regulates the osteogenesis of hASCs by influencing α-tubulin expression and Eg5 activity

**DOI:** 10.1016/j.gendis.2024.101380

**Published:** 2024-07-26

**Authors:** Tingyu Fan, Wenqing Liu, Rongmei Qu, Jinhui Zhu, Yulian Shi, Jiaxuan Liu, Xiangtian Li, Zhitao Zhou, Yunbing Chang, Jun Ouyang, Jingxing Dai

**Affiliations:** a Guangdong Provincial Key Laboratory of Digital Medicine and Biomechanics & Guangdong Engineering Research Center for Translation of Medical 3D Printing Application & National Virtual & Reality Experimental Education Center for Medical Morphology (Southern Medical University) & National Key Discipline of Human Anatomy, School of Basic Medical Sciences, Southern Medical University, Guangzhou, Guangdong 510515, China; b Central Laboratory, Southern Medical University, Guangzhou, Guangdong 510515, China; c Department of Spine Surgery, Guangdong Provincial People's Hospital (Guangdong Academy of Medical Sciences), Southern Medical University, Guangzhou, Guangdong 510515, China

Seed cells, scaffold materials, and growth factors constitute the three fundamental components of bone tissue engineering.^1^ In recent years, human adipose-derived stem cells (hASCs) have emerged as promising candidate cells owing to their several sources and strong differentiation ability.^2^ Therefore, studying the mechanisms underlying the osteogenesis of hASCs is crucial for further progress in bone tissue engineering. With the aid of bioinformatics, we conducted a comparative analysis of the differentially expressed genes between osteogenically induced and uninduced groups and identified a crucial gene, Eg5. Eg5, alternatively referred to as KIF11 or Eg5 kinesin motor protein, is a constituent of the kinesin motor protein family, exerting a substantial influence on the initial phases of cellular division through its regulation of spindle formation and functionality.^3^, ^4^, ^5^ Previous literature has suggested that Eg5 could generate the sliding force on the spindle pole microtubules, pushing the two extremes of the spindle away from each other and aiding in the accurate separation of chromosomes into daughter cells.^5^ Our team has long focused on the changes in the cytoskeletons in osteogenesis and found that microfilaments and microtubules are closely related to the osteogenesis of hASCs. Based on these findings, we hypothesized that Eg5, microtubules, and actin microfilaments might cooperate to regulate the osteogenesis of hASCs. Therefore, the present study focused on determining a potential regulatory association among Eg5, microtubules, and actin microfilaments and the direction of signal transduction across these elements. These findings will contribute novel perspectives and methodologies to inform future investigations in the field of bone tissue engineering.

The GSE75433 dataset was used for reanalysis. There were a total of 1546 differentially expressed genes, with 884 up-regulated and 662 down-regulated genes identified (Fig. S1A–D). GO network diagram revealed a close relationship between nuclear fission, organelle fission, and mitotic nuclear division (Fig. 1A; Fig. S1E–H and Table S1, 2). The protein–protein interaction network consists of 1451 nodes and 18,568 edges, and a core network with 127 nodes and 6834 edges was obtained via the MODE plugin (Fig. S1I, J). Through the cytoHubba plugin, we obtained ten key genes, forming a core network with 10 nodes and 45 edges (Fig. S1K). The protein–protein interaction network analysis showed that five genes, *KIF11*, *NUSAP1*, *AURKB*, *BUB1B*, and *TTK*, were simultaneously enriched in spindle nuclear division and organelle fission (Fig. 1B; and Table S3). In the HPA database, we found a higher Eg5 expression in bone marrow than in adipose tissue (Fig. S2A–C; and Table S4). The immunofluorescence staining of A-431, Rh30, and U2OS cells showed that Eg5 primarily expressed in the cytoplasm and colocalized with α-tubulin (Fig. S2D). Based on the findings, it is plausible to suggest a regulatory link between Eg5 and microtubules during osteogenesis.

**Figure 1:**
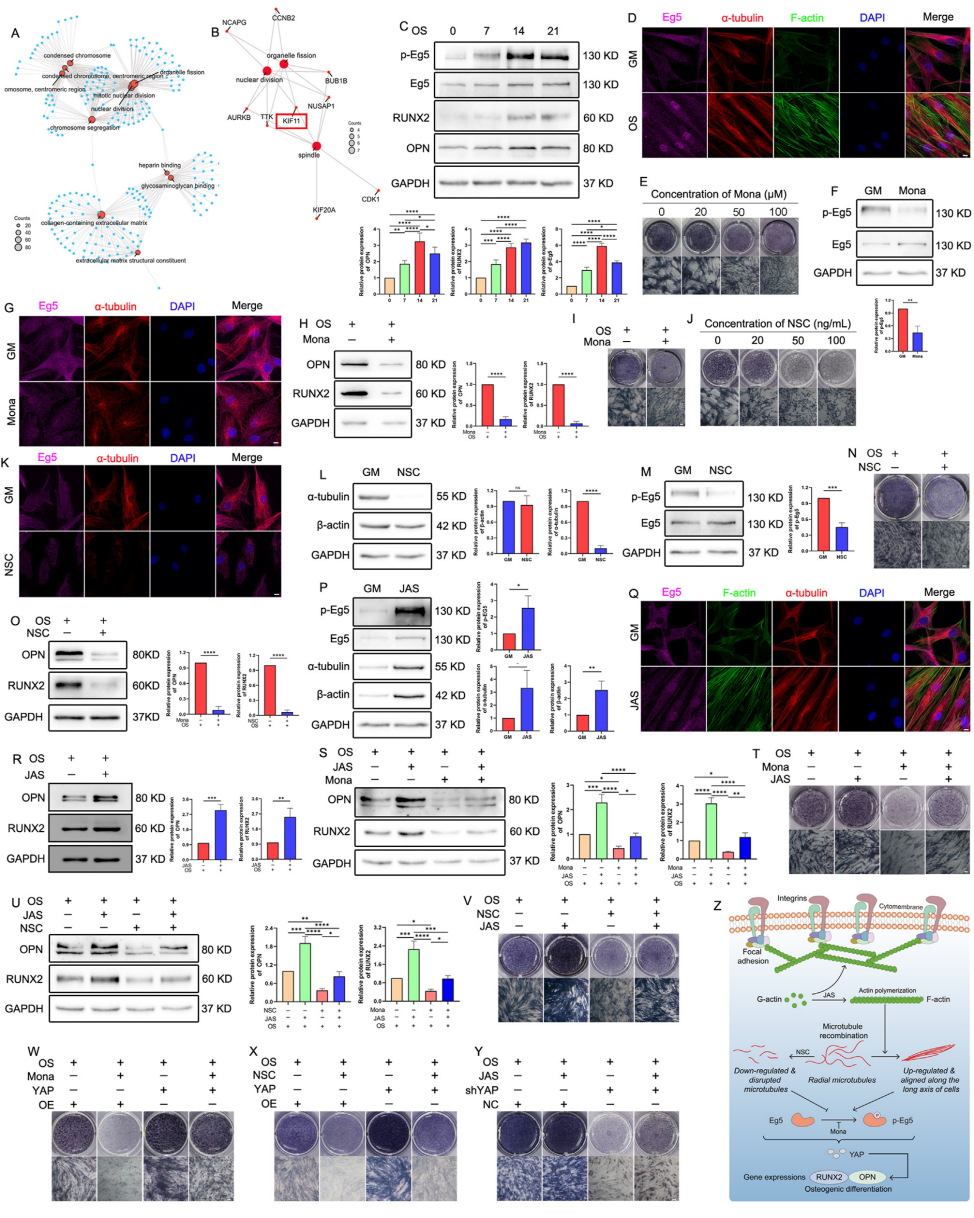
Actin polymerization regulates the osteogenesis of human adipose-derived stem cells (hASCs) by influencing α-tubulin expression and Eg5 activity. **(A)** The network analysis of GO terms. **(B)** The network of the top three enriched terms of hub genes. **(C)** The expression of OPN, RUNX2, Eg5, and p-Eg5 proteins was detected using a Western blot (WB) assay. **(D)** The morphologies of F-actin, α-tubulin, and Eg5 were observed using immunofluorescence. Scale bar, 20 μm. **(E)** ALP staining. 0, 20, 50, and 100 represent 0, 20, 50, and 100 μM, respectively. Scale bar, 100 μm. **(F)** The expression of Eg5 and p-Eg5 proteins was detected using WB assay. **(G)** The morphologies of Eg5 and microtubules were observed using immunofluorescence staining. Scale bar, 20 μm. **(H)** The expression of OPN and RUNX2 was detected via WB. **(I)** ALP staining. Scale bar, 100 μm. **(J)** ALP staining. 0, 20, 50, and 100 represent 0, 20, 50, and 100 ng/mL, respectively. Scale bar, 100 μm. **(K)** The morphologies of Eg5 and microtubules were observed using immunofluorescence. Scale bar, 20 μm. **(L, M)** The expression of α-tubulin, β-actin, Eg5, and p-Eg5 proteins was detected via WB. **(N)** ALP staining. Scale bar, 100 μm. **(O)** The expression of OPN and RUNX2 proteins was detected via WB. **(P)** The expression of α-tubulin, β-actin, Eg5, and p-Eg5 proteins was detected using WB assay. **(Q)** The morphologies of F-actin, α-tubulin, and Eg5 were observed using immunofluorescence. Scale bar, 20 μm. **(R)** The expression of OPN and RUNX2 proteins was detected using WB. **(S)** The expression of OPN and RUNX2 proteins was detected using WB assay. **(T)** ALP staining. Scale bar, 100 μm. **(U)** The expression of OPN and RUNX2 proteins was detected via WB assay. **(V–Y)** ALP staining. Scale bar, 100 μm. **(Z)** The diagram showing the impact of actin microfilaments on the osteogenesis of hASCs via α-tubulin expression and Eg5 activity. 0, 7, 14, and 21 represent 0, 7, 14, and 21 days of osteogenesis, respectively. GAPDH was used as the internal reference for WB. For immunofluorescence assay, Eg5, α-tubulin, F-actin, and the nucleus (DAPI) are depicted in purple, red, green, and blue, respectively. GM stands for growth medium. OS stands for osteogenic differentiation medium. JAS stands for 20 nM jasplakinolide. Mona stands for 100 μM monastrol. NSC stands for 100 ng/mL nocodazole. OE stands for empty vector control. YAP represents YAP up-regulated cells. NC stands for negative control. shYAP represents YAP down-regulated cells. ∗*P* < 0.05, ∗∗*P* < 0.01, ∗∗∗*P* < 0.001, ∗∗∗∗*P* < 0.0001.

We observed progressively increasing ALP (alkaline phosphatase) staining intensity and up-regulated OPN (osteopontin) and RUNX2 (Runt-related transcription factor 2) expression during hASC osteogenesis (Fig. S3A; 1C), suggesting that the cells possessed competent osteogenic differentiation ability. The western blotting (WB) results indicated that Eg5 and p-Eg5 up-regulated during osteogenesis (Fig. 1C). Immunofluorescence staining showed that during osteogenesis, the number and strength of actin microfilaments enhanced, microtubule fibers gradually aligned along the long axis of cells, and the co-localization of Eg5 with microtubules gradually increased (Fig. 1D). CCK8 assay showed a significant suppression in cell viability after treatment with 100 μM monastrol (Mona, an inhibitor of Eg5) (Fig. S3B). The cells treated with 100 μM Mona (Mona group) exhibited lower ALP staining intensity than the control group (Fig. 1E). Therefore, 100 μM Mona was used for subsequent analyses. Immunofluorescence staining and WB revealed Ki67 down-regulation in the Mona group (Fig. S3C, D). Furthermore, WB showed a ∼0.44-fold higher p-Eg5 expression in the Mona group than in the control group (Fig. 1F). However, we did not observe any significant difference in the microtubules between the control and Mona groups (Fig. S3E; Fig. 1G). These findings indicate that Eg5 inhibition did not affect microtubules, namely that the signal transduction direction was not from Eg5 to microtubules. Furthermore, we observed down-regulation of OPN and RUNX2 (Fig. 1H) and a weaker ALP staining intensity in the OS + Mona group than in the OS group (Fig. 1I). These findings suggest that Eg5 inhibition could lead to decreased cell proliferation and impaired osteogenesis of hASCs.

A high α-tubulin expression was observed during the osteogenesis of hASCs (Fig. S4A). CCK8 assay revealed that nocodazole (NSC, a microtubule depolymerizer) treatment inhibited cell proliferation in a concentration-dependent manner (Fig. S4B). A gradual decrease was found in the ALP staining intensity of the NSC-treated cells with increasing drug concentration (Fig. 1J). Therefore, 20 ng/mL NSC was used for subsequent experiments. Immunofluorescence staining and WB assay showed Ki67 down-regulation in the NSC group (Fig. S4C, D). Immunofluorescence analyses revealed disrupted microtubules in the NSC group (Fig. 1K), and the WB assay showed a down-regulation of α-tubulin expression in the NSC group (Fig. 1L). The NSC group also exhibited down-regulation of p-Eg5, OPN, and RUNX2 and a weaker ALP staining intensity than the control group (Fig. 1M−O). These data suggest that the direction of signal transduction is from microtubules to Eg5, and that microtubule depolymerization leads to reduced Eg5 activity and osteogenesis of hASCs.

WB analyses revealed β-actin up-regulation during the osteogenesis of hASCs (Fig. S5A). Immunofluorescence staining and WB assay revealed Ki67 up-regulation in the JAS (jasplakinolide, a microfilament polymerizer) group (Fig. S5B, C). The JAS group exhibited up-regulation of β-actin, α-tubulin, and p-Eg5 (Fig. 1P). We observed an increase in the number and thickness of actin fibers and enhanced alignment of microtubule fibers along the long axis of the cells in the JAS group than the cells in the control group (Fig. 1Q). The JAS + OS group exhibited OPN and RUNX2 up-regulation (Fig. 1R) and a stronger ALP staining intensity than the OS group (Fig. S5D).

Rescue experiments showed that OPN and RUNX2 expression in the cells treated with both Mona and JAS were higher and lower than in the cells treated with only Mona and only JAS, respectively (Fig. 1S). ALP staining experiments showed that the staining intensity in the cells treated with both Mona and JAS was stronger and weaker than in the cells treated with only Mona and only JAS, respectively (Fig. 1T). Eg5 inhibition attenuates the enhancement of osteogenesis of hASCs via actin polymerization. Besides, OPN and RUNX2 expression in the cells treated with both NSC and JAS were higher and lower than in the cells treated with only NSC and only JAS, respectively (Fig. 1U). ALP staining experiments showed that the staining intensity in the cells treated with both NSC and JAS was stronger and weaker than in the cells treated with only NSC and only JAS, respectively (Fig. 1V). Subsequently, we observed that the expression of YAP (Yes-associated protein) and TAZ (transcriptional co-activator with PDZ-binding motif) decreased when the activity of Eg5 was inhibited or microtubules were depolymerized (Fig. S6A, B). However, we only observed a significant up-regulation of YAP during actin polymerization, not TAZ (Fig. S6C). Therefore, we consider YAP to be a common downstream molecule of actin, microtubules, and Eg5. We further validated this result by manipulating the expression of YAP and conducting rescue experiments (Fig. S6D, E; Fig. 1W–Y). Therefore, we conclude that the signaling direction of osteogenesis of hASCs extended from actin microfilaments to microtubules to Eg5, indicating that actin polymerization regulates the osteogenesis of hASCs by impacting α-tubulin expression and Eg5 activity (Fig. 1Z).

## Ethics declaration

This study was approved by the Ethics Committee of the School of Basic Medical Sciences, Southern Medical University (No. NFYKDXJCYXY2016031002).

## Author contributions

Jingxing Dai, Jun Ouyang, and Yunbing Chang conceived and designed the experiments. Tingyu Fan, Wenqing Liu, Rongmei Qu, Jinhui Zhu, and Yulian Shi performed the experiments. Jiaxuan Liu, Xiangtian Li, and Zhitao Zhou analyzed the data. Tingyu Fan and Yulian Shi wrote the manuscript.

## Conflict of interests

The authors declared no conflict of interests.

## Funding

This study was financially supported by the National Key R&D Program of China (No. 2022YFF1202603) and the President's Foundation of TCM-Integrated Hospital of Southern Medical University (No. 1202103001).
