# Supplementary material for: Actin polymerization regulates the osteogenesis of hASCs by influencing α-tubulin expression and Eg5 activity
Source: Genes Dis. 2024 Jul 26;12(2):101380. doi: 10.1016/j.gendis.2024.101380 (PMC11585723; doi:10.1016/j.gendis.2024.101380)
Supplement: Multimedia component 1 [file mmc1.docx]

**Supplementary Figure Legends.**

**
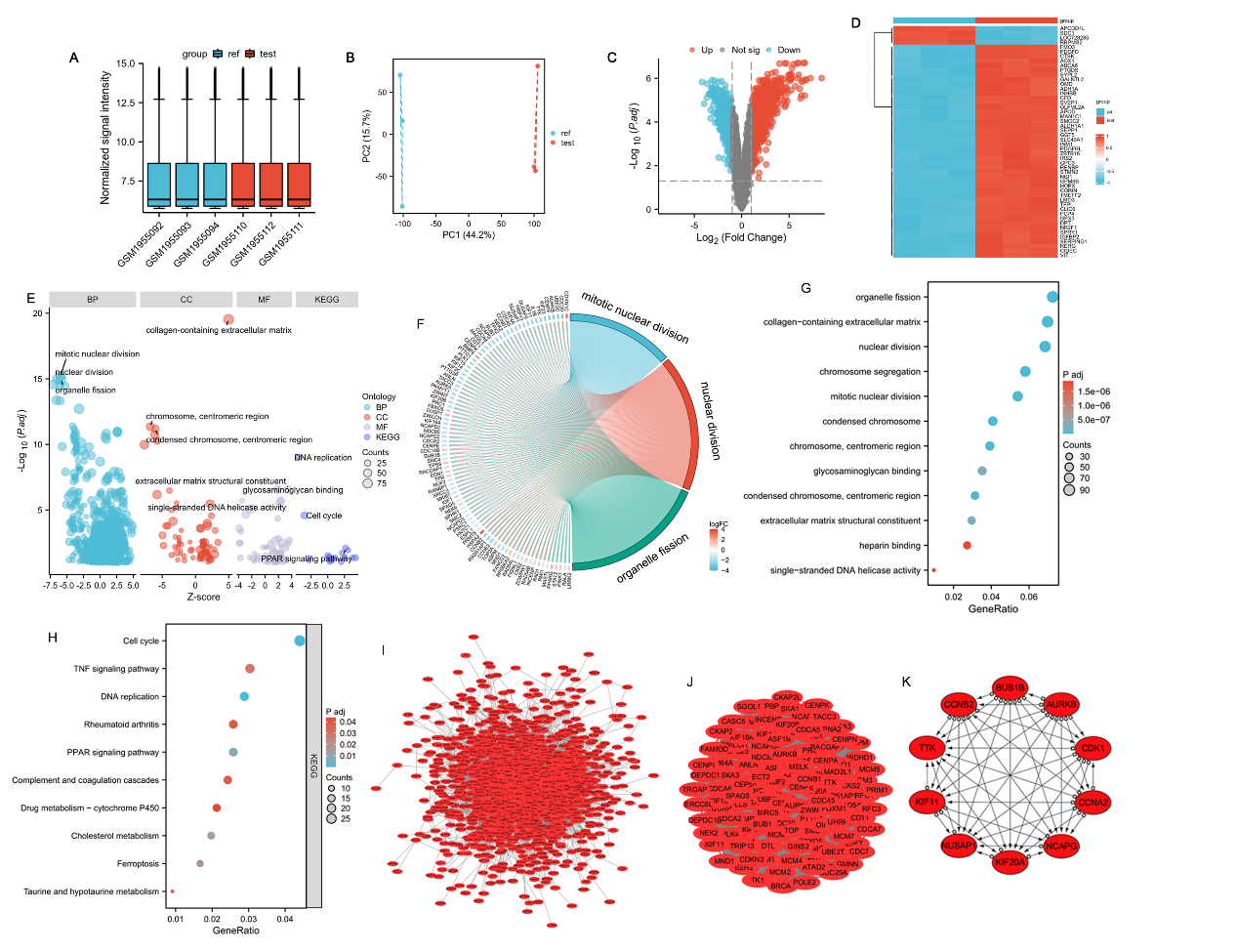
**

**Figure S1 Functional enrichment analysis of DEGs.** (**A**) box plot. (**B)** PCA diagram. (**C**) Volcano plot. (**D**) Heat map. *P* < 0.05, | logFC | > 1. Red dots represent up-regulated genes, and green dots represents down-regulated genes. Bubble diagram (**E**), and circle diagram (**F**) showing GO/KEGG enrichment analysis of DEGs association logFC. Ref: cells treated with growth medium. Test: cells treated with osteogenic differentiation medium. (**G**) Dot diagram showing the GO enrichment analyses of differentially expressed genes (DEGs). (**H**) Dot diagram showing KEGG enrichment analyses of DEGs. (**I**) The protein-protein interaction (PPI) network of DEGs. (**J**) The densest connected regions (127 nodes and 6834 edges) in the PPI network were identified using Cytoscape. (**K**) Ten hub genes. The score is indicated in red color.


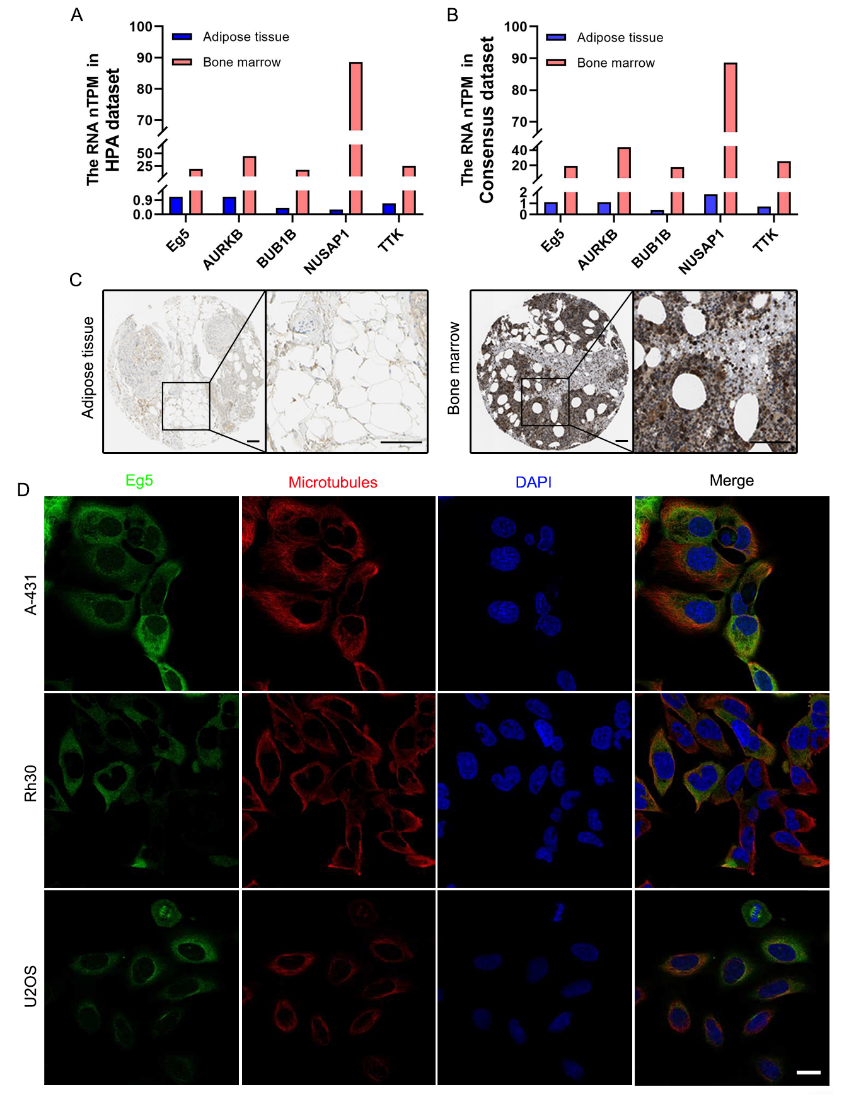


**Figure S2. Eg5 expression in human tissues.** (**A–B**) Eg5 mRNA expression levels in adipose tissue and bone marrow. The data were obtained from the HPA database. (**C**) Eg5 protein expression in adipose tissue and bone marrow, as detected by immunohistochemical staining. The data were obtained from the HPA database. Scale bar, 100 μm. (**D**) Immunofluorescence staining of the Eg5 protein in A-431, Rh30, and U2OS cells. The data were obtained from the HPA database. Scale bar, 20 μm.


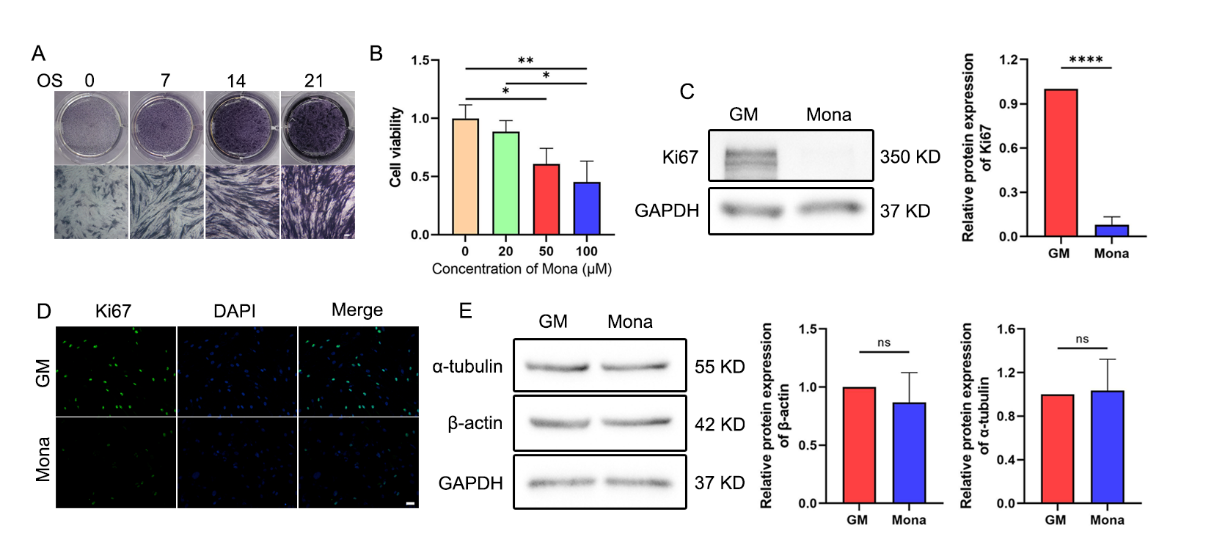


**Figure S3 Inhibition of Eg5 resulted in decreased cell proliferation.** (**A**) ALP staining. Scale bar, 100 μm. 0, 7, 14, and 21 represent 0, 7, 14, and 21 days of osteogenesis, respectively. (**B**) Cell viability was detected by CCK8 assay. 0, 20, 50, and 100 represent 0, 20, 50, and 100 μM, respectively. (**C**) WB assay was used to determine the expression level of Ki67 protein. (**D**) Ki67 expression was detected using immunofluorescence staining. Ki67 and the nucleus (DAPI) are depicted in green and blue, respectively. Scale bar, 200 μm. (**E**) The expressions of α-tubulin and β-actin proteins were detected using WB assay. GAPDH as the internal reference. GM stands for growth medium. Mona stands for growth medium containing 100 μM Mona. **P* < 0.05, ***P* < 0.01, ****P* < 0.001, *****P* < 0.0001.

**
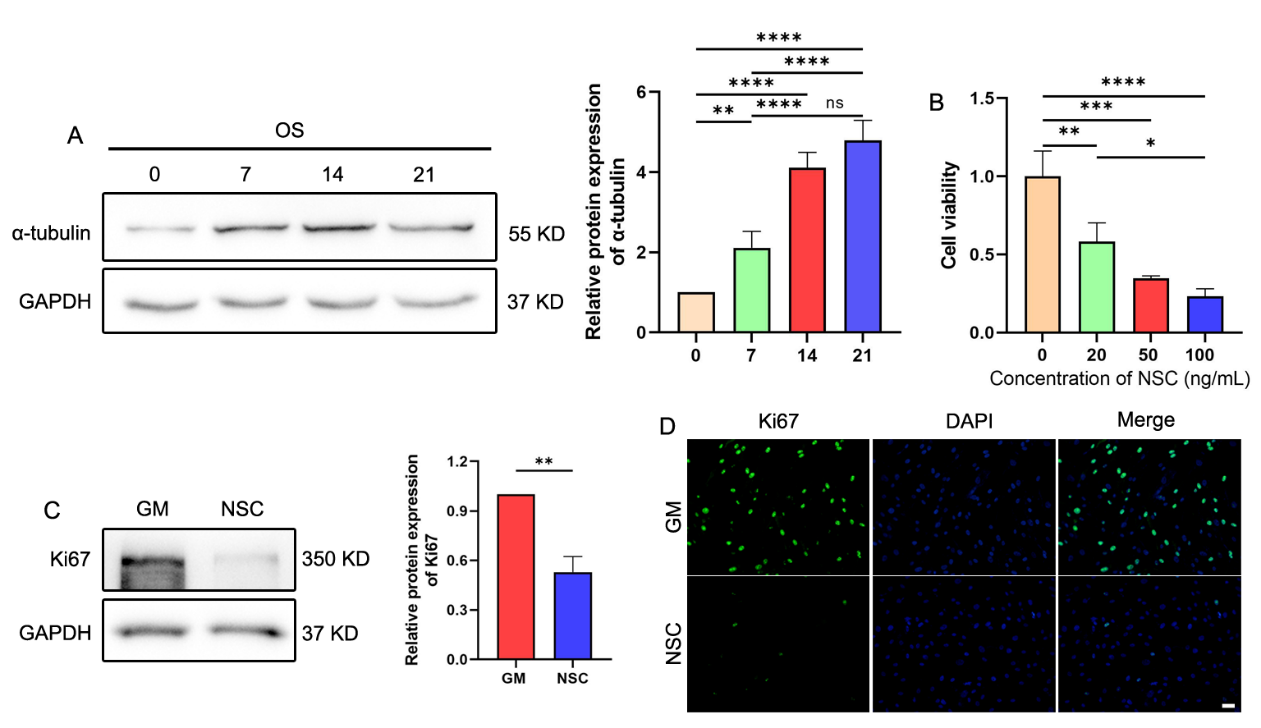
 Figure S4 Depolymerization of microtubules resulted in decreased cell proliferation.** (**A**) α-tubulin protein expression was detected via WB assay. 0, 7, 14, and 21 represent 0, 7, 14, and 21 days of osteogenesis, respectively. (**B**) Cell viability was detected by CCK8 assay. 0, 20, 50, and 100 represent 0, 20, 50, and 100 ng/mL, respectively. (**C**) WB assay was used to determine the expression level of Ki67 protein. (**D**) Ki67 expression was detected via immunofluorescence. Ki67 and the nucleus (DAPI) are depicted in green and blue, respectively. Scale bar, 200 μm. GAPDH as the internal reference. GM stands for growth medium. NSC stands for 20 ng/mL nocodazole. **P* < 0.05, ***P* < 0.01, ****P* < 0.001, *****P* < 0.0001.


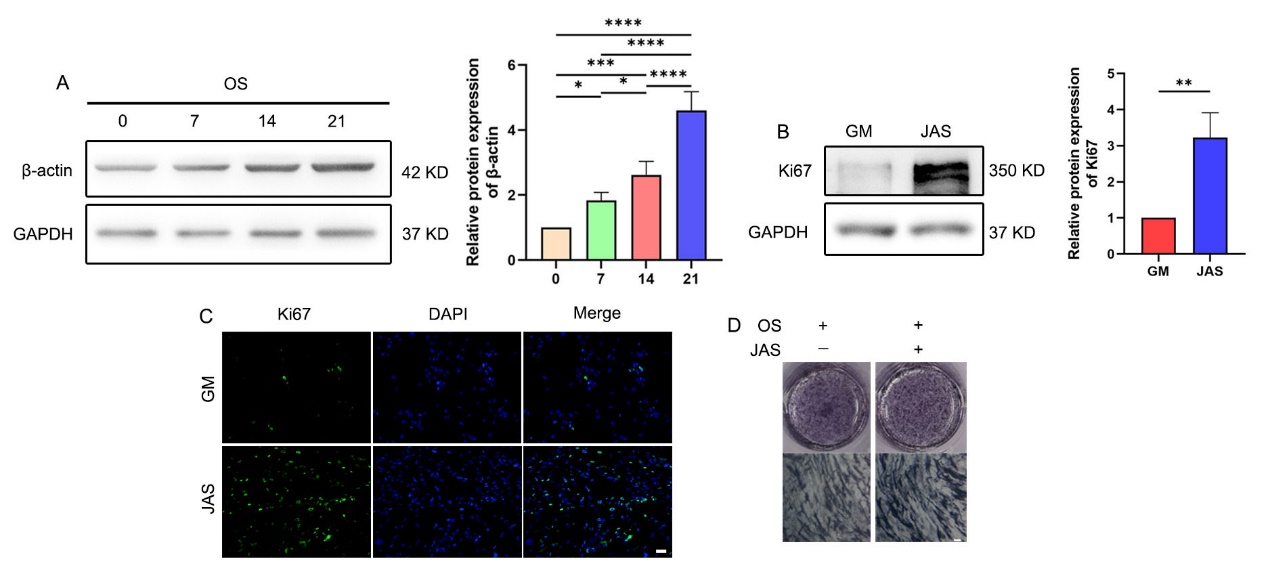


**Figure S5 Effect of microfilament polymerization on cell proliferation.** (**A**) β-actin protein expression was detected by WB assay. 0, 7, 14, and 21 represent 0, 7, 14, and 21 days of osteogenesis, respectively. (**B**) WB assay was used to determine the expression level of Ki67 protein. (**C**) Ki67 expression was detected via immunofluorescence. Ki67 and the nucleus (DAPI) are depicted in green and blue, respectively. Scale bar, 200 μm. (**D**) ALP staining. Scale bar, 100 μm. GAPDH as the internal reference. GM stands for growth medium. JAS stands for 20 nM Jasplakinolide. **P* < 0.05, ***P* < 0.01, ****P* < 0.001, *****P* < 0.0001.


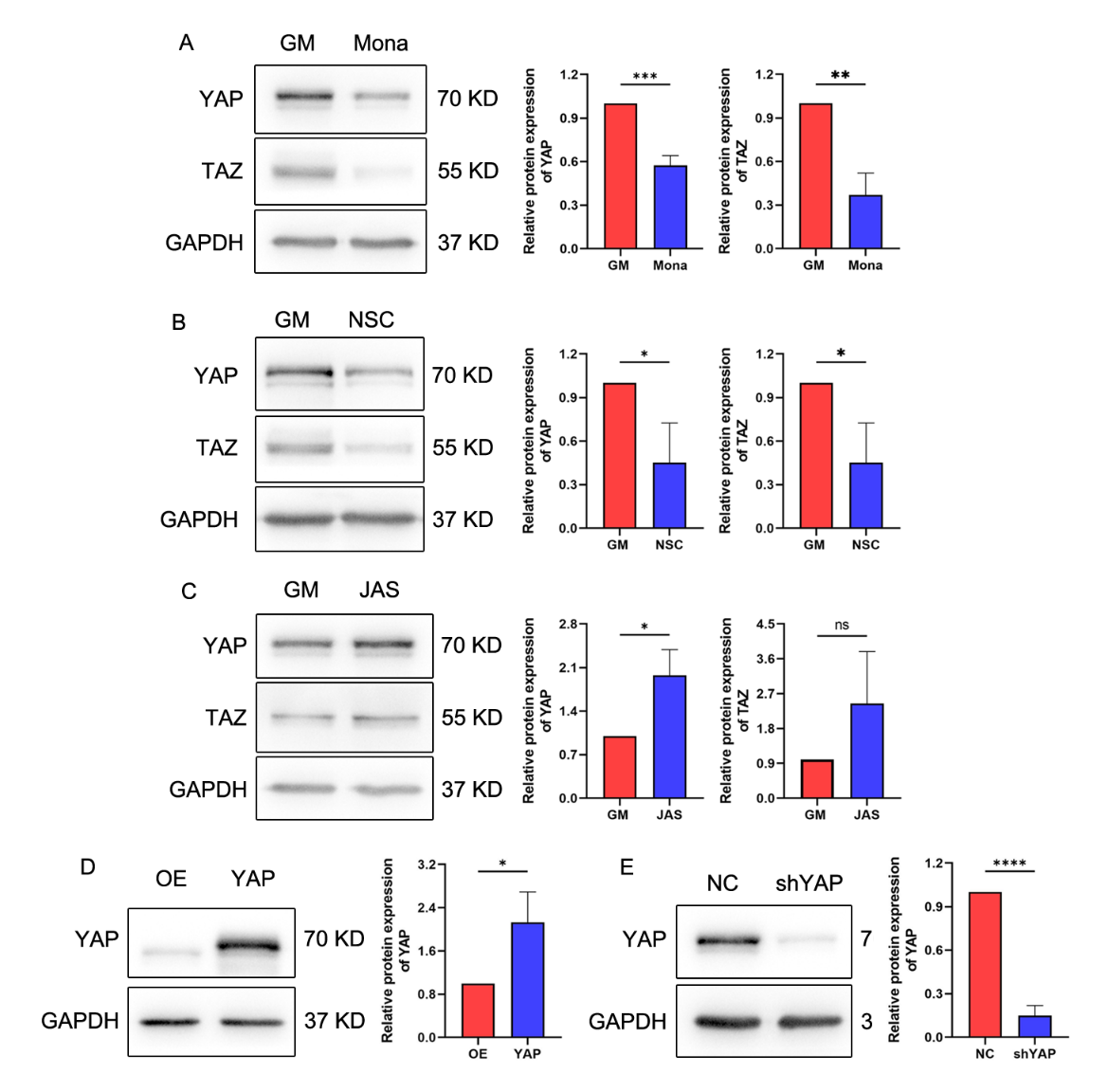


**Figure S6 YAP acts as a downstream effector of Eg5.** (**A-E**) The expressions of YAP and TAZ proteins were detected using WB assay. GAPDH was used as the internal reference. GM stands for growth medium. JAS stands for 20 nM jasplakinolide. Mona stands for 100 μM monastrol. NSC stands for 100 ng/mL nocodazole. OE stands for empty vector control. YAP represents YAP up-regulated cells. NC stands for negative control. shYAP represents YAP down-regulated cells. **P* < 0.05, ***P* < 0.01, ****P* < 0.001, and *****P* < 0.0001.
