# Supplementary material for: Actin polymerization regulates the osteogenesis of hASCs by influencing α-tubulin expression and Eg5 activity
Source: Genes Dis. 2024 Jul 26;12(2):101380. doi: 10.1016/j.gendis.2024.101380 (PMC11585723; doi:10.1016/j.gendis.2024.101380)
Supplement: Multimedia component 2 [file mmc2.docx]

| Ontology | ID | Description | GeneRatio | BgRatio | pvalue | p.adjust |
| --- | --- | --- | --- | --- | --- | --- |
| BP | GO:0140014 | mitotic nuclear division | 67/1241 | 293/18800 | 1.14e-19 | 6.48e-16 |
| BP | GO:0000280 | nuclear division | 85/1241 | 446/18800 | 3.48e-19 | 9.91e-16 |
| BP | GO:0048285 | organelle fission | 90/1241 | 493/18800 | 5.71e-19 | 1.08e-15 |
| BP | GO:0007059 | chromosome segregation | 72/1241 | 348/18800 | 1.88e-18 | 2.68e-15 |
| BP | GO:0000070 | mitotic sister chromatid segregation | 48/1241 | 171/18800 | 2.76e-18 | 3.15e-15 |
| CC | GO:0062023 | collagen-containing extracellular matrix | 89/1275 | 429/19594 | 4.91e-23 | 2.93e-20 |
| CC | GO:0000775 | chromosome, centromeric region | 50/1275 | 227/19594 | 1.49e-14 | 4.44e-12 |
| CC | GO:0000779 | condensed chromosome, centromeric region | 40/1275 | 156/19594 | 3.24e-14 | 6.45e-12 |
| CC | GO:0000793 | condensed chromosome | 52/1275 | 255/19594 | 1.18e-13 | 1.77e-11 |
| CC | GO:0000776 | kinetochore | 37/1275 | 146/19594 | 4.38e-13 | 5.23e-11 |
| MF | GO:0005201 | extracellular matrix structural constituent | 37/1251 | 172/18410 | 2.73e-10 | 2.7e-07 |
| MF | GO:0005539 | glycosaminoglycan binding | 44/1251 | 234/18410 | 5.83e-10 | 2.88e-07 |
| MF | GO:0017116 | single-stranded DNA helicase activity | 12/1251 | 23/18410 | 6.17e-09 | 1.87e-06 |
| MF | GO:0008201 | heparin binding | 34/1251 | 168/18410 | 7.56e-09 | 1.87e-06 |
| MF | GO:1901681 | sulfur compound binding | 45/1251 | 267/18410 | 1.32e-08 | 2.62e-06 |

**Table S1 GO enrichment analysis of DEGs.**
