# Supplementary material for: Actin polymerization regulates the osteogenesis of hASCs by influencing α-tubulin expression and Eg5 activity
Source: Genes Dis. 2024 Jul 26;12(2):101380. doi: 10.1016/j.gendis.2024.101380 (PMC11585723; doi:10.1016/j.gendis.2024.101380)
Supplement: Multimedia component 3 [file mmc3.docx]

**Table S2 KEGG enrichment analysis of DEGs.**

| Ontology | ID | Description | GeneRatio | BgRatio | pvalue | p.adjust |
| --- | --- | --- | --- | --- | --- | --- |
| KEGG | hsa03030 | DNA replication | 19/659 | 36/8164 | 3.09e-12 | 9.77e-10 |
| KEGG | hsa04110 | Cell cycle | 29/659 | 126/8164 | 1.65e-07 | 2.61e-05 |
| KEGG | hsa03320 | PPAR signaling pathway | 17/659 | 75/8164 | 7.41e-05 | 0.0078 |
| KEGG | hsa04979 | Cholesterol metabolism | 13/659 | 51/8164 | 0.0001 | 0.0116 |
| KEGG | hsa04216 | Ferroptosis | 11/659 | 41/8164 | 0.0003 | 0.0184 |
