# Supplementary material for: Actin polymerization regulates the osteogenesis of hASCs by influencing α-tubulin expression and Eg5 activity
Source: Genes Dis. 2024 Jul 26;12(2):101380. doi: 10.1016/j.gendis.2024.101380 (PMC11585723; doi:10.1016/j.gendis.2024.101380)
Supplement: Multimedia component 4 [file mmc4.docx]

**Table S3 Functional enrichment analysis of hub genes.**

| Term | Description | Count in gene set | p.adjust | Gene symbol |
| --- | --- | --- | --- | --- |
| GO:0005819 (CC) | spindle | 7 | 5.62142E-09 | *CDK1*, *KIF20A,* *AURKB*, *KIF11,* *BUB1B*, *TTK*, *NUSAP1* |
| GO:0000280 (BP) | nuclear division | 7 | 1.68068E-07 | *AURKB*, *NCAPG*, *KIF11*, *BUB1B*, *TTK*, *NUSAP1*, *CCNB2* |
| GO:0048285 (BP) | organelle fission | 7 | 1.69081E-07 | *AURKB,* *NCAPG*, *KIF11*, *BUB1B*, *TTK*, *NUSAP1*, *CCNB2* |
| GO: Gene Ontology; BP: biological processes; CC: cellular components. | | | | |
