# Supplementary material for: Actin polymerization regulates the osteogenesis of hASCs by influencing α-tubulin expression and Eg5 activity
Source: Genes Dis. 2024 Jul 26;12(2):101380. doi: 10.1016/j.gendis.2024.101380 (PMC11585723; doi:10.1016/j.gendis.2024.101380)
Supplement: Multimedia component 5 [file mmc5.docx]

**Table S4 Protein and RNA expressions of hub genes in HPA and Consensus database.**

| Gene name | Protein expression in HPA database (Score) | | RNA expression in Consensus database (nTPM) | | RNA expression in HPA database (nTPM) | |
| --- | --- | --- | --- | --- | --- | --- |
|  | Adipose tissue | Bone marrow | Adipose tissue | Bone marrow | Adipose tissue | Bone marrow |
| *KIF11* | Low | High | 1.1 | 19.4 | 1.1 | 19.4 |
| *TTK* | Not detected | Medium | 0.7 | 25.3 | 0.7 | 25.3 |
| *AURKB* | Low | Medium | 1.1 | 44.1 | 1.1 | 44.1 |
| *BUB1B* | no data | no data | 0.4 | 17.8 | 0.4 | 17.8 |
| *NUSAP1* | Not detected | High | 1.8 | 88.6 | 0.3 | 88.6 |
